# The convolutional neural network as a tool to classify electroencephalography data resulting from the consumption of juice sweetened with caloric or non-caloric sweeteners

**DOI:** 10.3389/fnut.2022.901333

**Published:** 2022-07-19

**Authors:** Gustavo Voltani von Atzingen, Hubert Arteaga, Amanda Rodrigues da Silva, Nathalia Fontanari Ortega, Ernane Jose Xavier Costa, Ana Carolina de Sousa Silva

**Affiliations:** ^1^Instituto Federal de São Paulo, Piracicaba, Brazil; ^2^Escuela Ingeniería de Industrias Alimentarias, Universidad Nacional de Jaén, Jaén, Peru; ^3^Departamento de Ciências Básicas, Faculdade de Zootecnia e Engenharia de Alimentos, Universidade de São Paulo, São Paulo, Brazil

**Keywords:** sucrose, sucralose, aspartame, electroencephalography (EEG), convolutional neural network (CNN)

## Abstract

Sweetener type can influence sensory properties and consumer’s acceptance and preference for low-calorie products. An ideal sweetener does not exist, and each sweetener must be used in situations to which it is best suited. Aspartame and sucralose can be good substitutes for sucrose in passion fruit juice. Despite the interest in artificial sweeteners, little is known about how artificial sweeteners are processed in the human brain. Here, we applied the convolutional neural network (CNN) to evaluate brain signals of 11 healthy subjects when they tasted passion fruit juice equivalently sweetened with sucrose (9.4 g/100 g), sucralose (0.01593 g/100 g), or aspartame (0.05477 g/100 g). Electroencephalograms were recorded for two sites in the gustatory cortex (i.e., C3 and C4). Data with artifacts were disregarded, and the artifact-free data were used to feed a Deep Neural Network with tree branches that applied a Convolutions and pooling for different feature filtering and selection. The CNN received raw signal as input for multiclass classification and with supervised training was able to extract underling features and patterns from the signal with better performance than handcrafted filters like FFT. Our results indicated that CNN is an useful tool for electroencephalography (EEG) analyses and classification of perceptually similar tastes.

## Introduction

Sugar has been the main sweetener in human diet for centuries. It represents a high percentage of the human daily energy consumption, but it offers little additional nutritional value (1–3).

Given that replacing sugar with non-caloric (or low-calorie) sweeteners have become popular among consumers seeking to lose or to maintain weight. Moreover, foods with the same sweetening capacity might be perceived differently due to their caloric content (4, 5), behavioral and physiological effects (6). Additionally, the brain might respond distinctly to perceptually similar and identical tastes (7).

Any study involving equi-intense sweeteners (i.e., sweeteners used in equivalent amounts) must consider how sweeteners influence sensory properties and consumer’s acceptance and preference for low-calorie products (8). An ideal sweetener does not exist, so each sweetener is appropriate for specific situations (9). Passion fruit is a popular tropical fruit that has an important commercial variety named yellow passion fruit, which is used to prepare juice that requires sweetening (10). Aspartame and sucralose can be good substitutes for sucrose in passion fruit juice (11, 12).

Gustatory stimuli are processed by the brain considering their physical properties and chemical qualities. The ability of electroencephalography (EEG) to access brain processes has already been used in studies of a sensory nature (7, 13–17). The EEG signal acquired with a single channel can be used to monitor mental activity (17–19). Experiments using infrared spectroscopy (20) and magnetoencephalography (MEG) (21, 22) have been carried out to locate possible taste areas of the cerebral cortex and to analyze differences between stimuli. Other methods, based on nonlinear dynamics, have shown that the taste of chewing gum alters the nonlinear characteristics and frequency domain of EEG (23).

In addition, from the food science standpoint, several aspects must be considered when it comes to taste perception, including the hypothesis that the taste perception threshold is related not only to the sensitivity of the sensory organ—in this case, the tongue—but also to a cognitive process in the brain (20, 24, 25).

Although EEG has been demonstrated to be a valuable tool for research in various applications, it has several limitations which affect analysis and processing performance. Because of their outstanding robustness and adaptability, several machine learning (ML) and deep learning (DL) models for performing EEG signal classification have been reported (26). DL is a new field of ML that allows computational methods composed of multiple processing layers to be employed (27) and Convolutional Neural Networks (CNNs) are a supervised learning approach. CNN were first proposed by Fukushima (27, 28), but this approach was not widely employed because computation hardware was limited for training the networks. Deep CNNs are typical feedforward neural networks to which Backpropagation (BP) algorithms are applied to adjust the network parameters (weights and biases), to reduce the cost function value (29).

Two types of layers exist in the network low- and middle-levels: convolutional layers and max-pooling layers. The output nodes of the convolution and max-pooling layers are grouped into a 2D plane called feature mapping. As the features propagate to the highest layer or level, the dimensions of features are reduced depending on the kernel size for the convolutional and max-pooling operations, respectively. These hidden layers allow nonlinear and complex problems to be handled (30, 31).

In this study, we employed CNN to classify EEG data resulting from the consumption of drinks (passion fruit juice) sweetened with caloric (sucrose) and non-caloric sweeteners (sucralose and aspartame). Our goal is to test the hypothesis that CNN is a useful tool to feature extraction and classification of EEG data resulting from stimulation with perceptually similar tastes.

## Materials and methods

The methods used herein were divided into three main sections: (1) Participant selection, (2) acquisition of EEG signal from the selected group, and (3) signal processing and Convolutional Neural Network (CNN) training and tests. Our aim was to use a CNN to determine differences between stimuli.

### Stimuli

The passion fruit juices were prepared in the laboratory; unsweetened passion fruit pulp (DeMarchi™) was employed. The following ratio was used: one part of pulp to two parts of water. The juice samples were sweetened with sucrose (União™), aspartame (Ajinomoto™), or sucralose. Pure water was considered as reference. The samples were prepared 1 day before the experiment, stored at 4–6°C, and tested at room temperature.

The sweetener concentration in the passion fruit juice was chosen according to Rocha and Bolini’s study (11, 12), who stated that 9.4/100 g was an ideal sucrose concentration according to consumer’s acceptance. They also stated that the equivalent aspartame and sucralose concentrations in passion fruit juice were 0.0547 g/100 g and 0.0159 g/100 g, respectively.

### Participant selection

A total of 105 volunteers were investigated in a single-blind session. The volunteers comprised students, teachers, and employees aged 19–55 years, recruited on campus. They did not have diabetes, smoke, or use medications that affect taste or cognitive processes. Preference was given to volunteers that were used to consuming passion fruit juice (or that at least had no aversion to the fruit taste).

Our objective was to select individuals with a perception of sugar ideal sweetness as close as possible to 9.4 g/100 g and, among these individuals, to select those that had good ability to order the samples according to the sugar concentration. Volunteers who met these conditions and agreed to participate in signal acquisition sessions were selected.

Each volunteer received samples containing 30 mL of passion fruit juice sweetened with different amounts of sugar (i.e., 4.7, 7.05, 9.4, 11.75, and 14.1 g). The samples were placed in disposable cups and numbered randomly between 0000 and 9999 (e.g., A7932). The participants received samples in a randomized sequence of concentrations and had to answer the following question: “How much sugar did you have in your juice?” Answers were collected in a 9.0-cm visual scale (VAS). VASs are input mechanisms that allow users to specify a value within a predefined range. The volunteers were instructed to consider the center of the scale as ideal sweetness, 0 as less sweet than ideal, and 9 as sweeter than ideal.

The volunteers were informed about the nature and aims of the experiments and provided informed consent. The study was approved by the Ethics Committee of the Animal Science and Food Engineering College (FZEA) of the University of São Paulo (USP) (CAAE 59017516.6.0000.5422).

### Electroencephalography recording

Once finished the participant selection study, 11 individuals (both sexes) agreed to participate in the following intervention study. In this sense, EEG signals were acquired from individuals while they were tasting passion fruit juice. The individuals had signed an informed consent form (ICF).

Electroencephalography was carried out non-invasively on the scalp surface; the participants wore an EEG cap. The signal was acquired at positions C3 and C4 in the primary gustatory cortex (17, 22) as defined by the international 10–20 electrode disposal system (32). This electrode position was chosen on the basis of the study by Kabayakawa et al. (22), who showed magnetic fields recorded from the brain (i.e., MEG) in response to two tastants: 1 M NaCl and 3 mM saccharin, one of which was more activated in the central area of the head at positions C3 and C4. The ground electrodes were placed on the participant’s ear lobes, and a reference electrode was placed on the forehead. The signals were sampled at 512 Hz by using an iCelera digital portable electroencephalograph. Each recording lasted 16 s.

The volunteers were accommodated inside a Faraday cage, and the EEG signal started being recorded when the volunteer drank the solution (juice). Subjects were investigated in three single-blind sessions separated by an interval of at least 1 day. Each volunteer received the samples (30 mL) in duplicate in the following order: water (reference), passion fruit juice sweetened with sucrose, passion fruit juice sweetened with sucralose, and passion fruit juice sweetened with aspartame. The samples were placed in disposable cups and numbered randomly between 0000 and 9999 (e.g., A7932). Each volunteer participated on three different days of the experiment (repetitions). In the intervals between the sweetened samples, the participants received sparkling water to clean residues from their palate and to reduce other interferences.

### Signal processing and convolutional neural network training

To illustrate the temporal dynamics of taste we averaged electrical activity (averaged across participants) in response to each stimuli (33).

The signal was processed by using the Python programming language and the Pandas, NumPy, and SciPy libraries. These libraries are employed to solve mathematical and scientific problems, including signal processing. Initially, the data were inspected, and the recordings with many artifacts were excluded from the database. The remaining data consisted of signals from 68 experiments lasting 16 seconds each, recorded in two channels (i.e., C3 and C4). These signals came from one of four stimuli (i.e., sucrose, sucralose, aspartame, or water) and were captured across 3 days of repetitions per volunteer. The data were bandpass-filtered from 8 to 40 Hz, and each signal was divided into 2-s segments with a 0.1 s stride. This means that 16-s segments (512 samples per second) were resampled in 9,520 vectors at a length of 1,028. The EEG dataset and the processing code that supported the findings of this study are available on GitHub.^1^

When the vectors with length 1,028 were used as inputs for the CNN, they were structured as a network consisting of three parallel convolution processes merged into a fully two-layer-deep connected network.

The deep learning model was built by using an open-source Python library, i.e., Keras, which runs in a TensorFlow background. Optimal performance (e.g., number of neurons on a given layer and number of filters and kernel size on the convolution layer) was determined by using manual fine adjustment of the hyperparameters (e.g., number of neurons per fully connected layer and number of filters and kernel size for convolutional layers).

The last network layer contained four neurons, which were responsible for mapping four possible results of an entry (water, sugar, aspartame, or sucralose). A categorical cross-entropy loss function was used.

Training was accomplished by using an Adam algorithm with two-thirds of the data, while the test was performed with the remaining one-third.

The problem presented here is a multiclass (water, sugar, aspartame, or sucralose) classification task. Results of classifier validation studies are often presented as confusion matrices. A confusion matrix for *k*-class classification is a *k* × *k* contingency table whose cells [*i,j*] (*i* = 1,…,*k*, *j* = 1,…,*k*) present frequencies of observations with real class *C*_*i*_ and inferred class *C*_*j*_. A binary confusion matrix is a special case when there are only two classes: C (positive class) and not-C (negative class). A *k* × *k* confusion matrix can always be represented as a set of *k* binary confusion matrices, one for each class *C*_*i*_.

In a binary confusion matrix, observations classified correctly into the positive class are called true positives and observations classified correctly into the negative class are called true negatives. Instances of the positive class classified falsely as negative are called false negatives and instances of negative class classified falsely as positive are called false positives. Numbers of true positive, false positive, true negative and false negative observations are notated by TP, FP, TN, and FN (34). From these frequencies, performance evaluation metrics, such as accuracy, precision, recall and F_1_ score were calculated as follows.


(1) $$A\,c\,c\,u\,r\,a\,c\,y=\frac{T\,P+T\,N}{T\,P+T\,N+F\,P+F\,N}$$

(2) $$P\,r\,e\,c\,i\,s\,i\,o\,n=\frac{T\,P}{T\,P+F\,P}$$

(3) $$R\,e\,c\,a\,l\,l=\frac{T\,P}{T\,P+F\,N}$$

(4) $$F_{1}\,1\,s\,c\,o\,r\,e=\frac{2\,\left(p\,r\,e\,c\,i\,s\,i\,o\,n\right)*\left(r\,e\,c\,a\,l\,l\right)}{\left(p\,r\,e\,c\,i\,s\,i\,o\,n\right)+\left(r\,e\,c\,a\,l\,l\right)}$$

where TP (true positive), FP (false positive), TN (true negative) and FN (false negative) are technical terms for binary classifier. Specific, TP is the positive samples correctly classified, FP is the positive samples misclassified, TN is the negative samples correctly classified and FN represents the negative samples misclassified. These performance indicators reflect how the classifier performs in detecting the given class.

## Results and discussion

### Participant selection

We selected the groups according to two criteria, namely, preference for the sample with a sucrose concentration of 9.4 g/100 g and good ability to order the sample concentrations. This means that the selected participants indicated values around 4.5 cm on the VAS, which was equivalent to a sucrose concentration of 9.4 g/100 g. Furthermore, they were able to order all the concentrations properly. Of the individuals considered fit, 11 agreed to participate in the brain signal acquisition stage.

**FIGURE 1:**
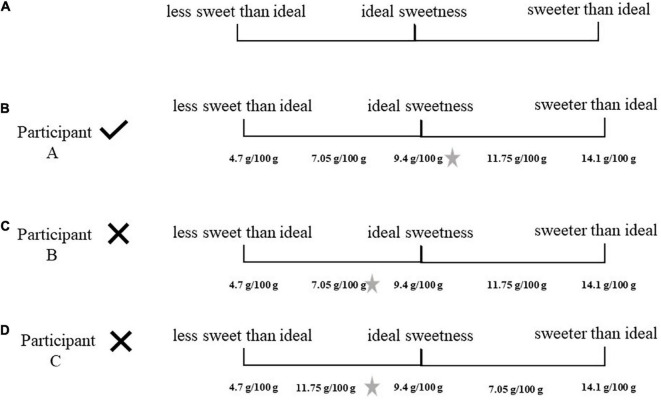
**(A)** Participant selection form. **(B)** Participant selection form from a selected participant. **(C)** Participant selection form from a discarded participant. **(D)** Participant selection form from a discarded participant. The star placed on the right side illustrates participant preferred sample. In the original form, the volunteer marked the sample number on the scale. For didactic purposes, these codes were replaced with the sample concentration in the figure.

Figure 1A illustrates the scale presented to the participants. Figure 1B shows participant A, who placed all the samples in the correct sequence of concentrations and chose the sample with sugar concentration of 9.4 g/100 g as his favorite. He was selected for the next step. Participants B and C were not selected. Participant B (Figure 1C) placed the samples correctly, but his preferred sample was not the sample with sugar concentration of 9.4 g/100 g. Although participant C (Figure 1D) preferred the sample with a sugar concentration of 9.4 g/100 g, he was not able to order the samples correctly.

### Signal processing and convolutional neural network training

**FIGURE 2:**
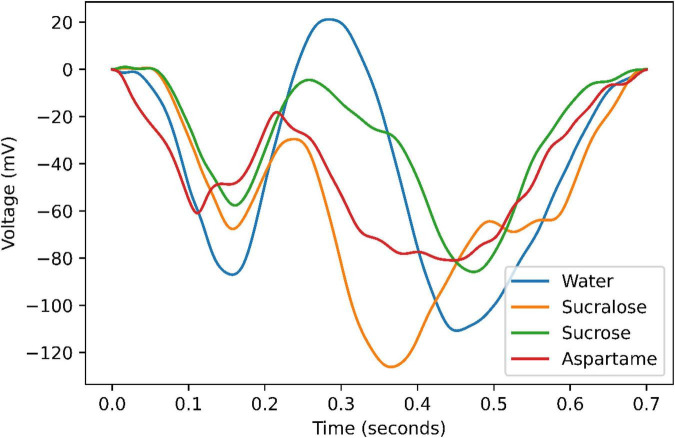
Signal strength quantified as the average within-subjects over two electrodes for each of the tastants and water.

Brain responses to the three taste stimuli (sucrose, sucralose, and aspartame) and reference in temporal domain are presented in Figure 2. As expected, (7) the activity of taste responses increases after stimuli onset and decreases again.

**FIGURE 3:**
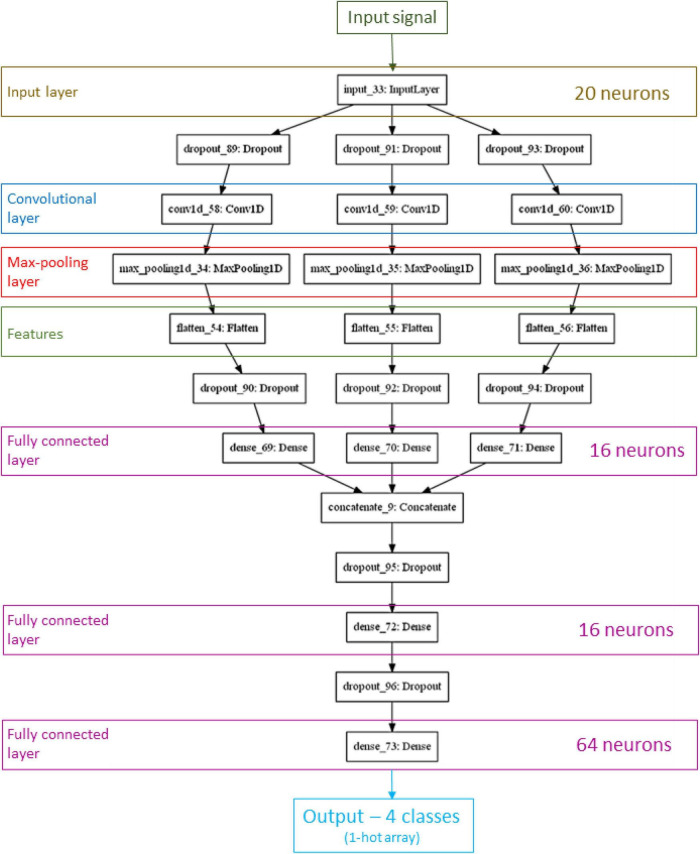
The convolutional neural network (CNN) architecture that achieved the best performance.

Figure 3 shows the network architecture that achieved the best performance. It is structured as a network consisting of three parallel convolution and max-pooling processes. Convolutional layer output is a feature map that has its dimension reduced by pooling layers. Each branch produces a different pattern array. Theses outputs are merged into a fully two-layer-deep connected network. An additional layer connects this combined result to output neurons (one for each class). To reduce overfitting all these processes are followed by dropout operation. The best number of neurons in input layer (*n*) was 20, as can be seen by the input layer shape. The other three fully connected hidden layers had 16, 16, and 64 neurons. Its dropout optimal value was 0.2. We used this optimal architecture to train the CNN with 70% of the data, while we employed the remaining 30% for the test.

**FIGURE 4:**
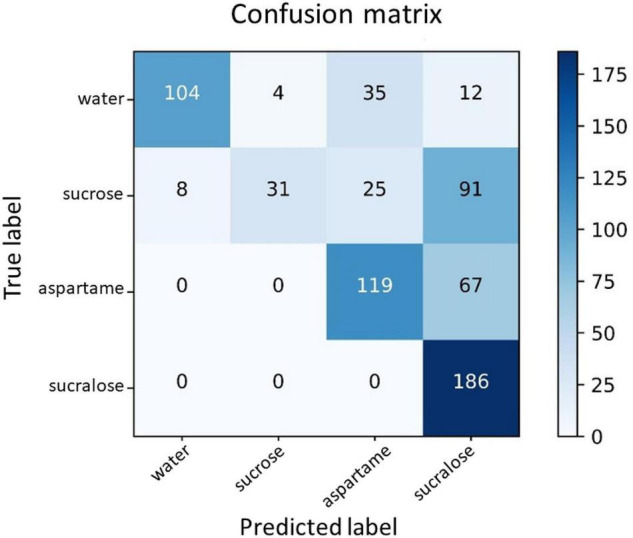
Confusion matrix for the classes water, sucrose, sucralose, and aspartame. The horizontal axis is the predicted label, and the vertical axis is the true label. The elements on the diagonal represent the numbers of correctly classified samples.

For visualization, Figure 4 presents the confusion matrix for this dataset. In the confusion matrix, the horizontal axis is the predicted label, and the vertical axis is the true label. The elements on the diagonal represent the numbers of correctly classified samples.

In Figure 4, for the water class, 104 samples were correctly predicted as water (67.1%), whereas 35 samples (22.6%) were incorrectly predicted as aspartame and four samples (2.6%) were incorrectly predicted as sucralose. For the sucrose class, 31 samples (20.0%) were correctly predicted as sucrose, whilst 25 samples (16.1%) were incorrectly predicted as aspartame, and 91 samples (58.7%) were incorrectly predicted as sucralose. As for the aspartame class, 119 samples (64.0%) were correctly classified, while 67 samples (36.0%) were incorrectly predicted as sucralose. Concerning the sucralose class, 186 samples (100.0%) were correctly classified. The main difficulty lay in the classes of water and sucrose.

Table 1 lists the metrics results for the four stimuli (classes). As a result, the average metrics were: 0.823 for accuracy, 0.750 for precision, 0.628 for recall, and 0.611 for f_1_ score. There are no studies using CNN in similar databases for comparison purposes, but these values are compatible with those obtained for studies involving brain signals to assess emoticons (35) and cerebral dominance (36).

**TABLE 1:** Results of the convolutional neural network (CNN) classification performance for the four stimuli (classes).

	Accuracy	Precision	Recall	F_1_ score
Water	0.9135	0.9286	0.6710	0.7790
Sucrose	0.8123	0.8857	0.2000	0.3263
Aspartame	0.8138	0.6648	0.6398	0.6521
Sucralose	0.7507	0.5225	1.0000	0.6863

When we consider reference (water) only, the classification in Figure 3 indicated that the average identification accuracy is 91.4% compared to an overall classification of 82.3%. This work uses a drink instead of a solution and, in a first study of this nature, it was important to be able to distinguish a critical reference well.

Analyzing Figure 4 in more detail, suggests that the network is more sensitive to the low-calorie sweetener classes than to the sucrose class. By evaluating the false positives (FP) of the classification, aspartame can be predicted to be sucralose, but not sugar. In turn, sucralose had no false positives. The most surprising result is that sucrose can be classified both as sugar and low-calorie sweetener. In other words, evaluated low-calorie sweeteners had not been confused with the caloric sweetener, but the caloric sweetener can be confused with the low-calorie sweetener. The explanation for this result may be a combination of different factors that are able to influence gustation, as type of tongue receptor, reward system, preference, and history of consumption of non-caloric sweeteners.

Sweet sensation activates areas of the brain involved in food memory and reward, but various sweet compounds differ in their specific effects (6). Frank et al. (5) observed that only sucrose, but not sucralose, stimulation engages dopaminergic midbrain areas in relation to the behavioral pleasantness response and states that this may suggest that sucralose activates taste reward circuits but may not fully satisfy a desire for natural caloric sweet ingestion.

Many artificial sweeteners have an aftertaste that is easily detectable, which may induce a cognitive or affective bias toward the substance ingested. Sucralose has a little bitter after taste reported (6), in opposite to aspartame that has a prolonged aftertaste response (7, 37). This may be one of the reasons why low-calorie sweeteners were not misclassified as sugar.

Andersen et al. (7) observed that similar tastes that are consciously indistinguishable can result in different brain cortical activations. A similar result was obtained when gustatory evoked potentials (GEPs) were used to assess the brain response to sucrose, aspartame, and stevia in humans (14). The authors stated that, although sucrose, aspartame, and stevia led to the same taste perception, the GEPs showed that cerebral activation by these different sweet solutions had different recordings. They suggested that, besides the difference in taste receptors and cerebral areas activated by these substances, neural plasticity and changes in the synaptic connections related to sweet innate preference and sweet conditioning could explain the differences in cerebral gustatory processing after sucrose and sweetener activation. The results presented herein agree with what was pointed out by Andersen et al. (7).

Unlike Andersen et al. (7) and Mouillot et al. (14), in this study we evaluated a beverage (i.e., passion fruit juice) with good acceptance after it is sweetened with aspartame or sucralose (11, 12) as a stimulus instead of evaluating a sweet solution. This may explain FP rates in classification. During tasting, the stimuli came not only from the sweet solution, but also from the other sensory characteristics of the beverage. Passion fruit gives a juice with a very strong and acid exotic flavor (10) and this acid may be an additional factor for misclassification, since among the five basic taste qualities (sweet, bitter, umami, salty, and sour), acid or sour sensing is particularly unique because it is mediated not only by the taste system but also by the somatosensory system *via* Trpv1-expressing neurons innervating the oral cavity (38).

Although the sweeteners were chosen in equi-intense amounts (11, 12, 39), it is quite possible that the combination of the unique characteristics of passion fruit juice, with each of the sweeteners, including sugar, activates the reward systems and the individual’s memories in a different manner. This could be one of the factors that justify the FP in the sucrose classification.

Apart from using a beverage instead of a solution and from dispensing a taste delivery system (5, 7, 15, 21, 22) that is common in this kind of experimental design, we only placed two electrodes near the gustatory cortex. While this is not a new strategy (17–19), it is uncommon (5, 7, 15, 21, 22) and has advantages and disadvantages. The main disadvantage is that it reduces the amount of data and makes some feature extraction techniques unfeasible. On the other hand, developing commercial applications based on a single EEG channel, such as the applications used by Hashida et al. (17) and Silva et al. (40), is easy.

The process of food tasting starts from tongue, where different taste receptors respond to various taste stimuli and pass the signals to the cortex of the brain region. Several important factors influence the taste perception and brain response, as age, gender, ethnicity, habits, stimuli type, temperature, and state of mind (41). Monitoring brain activity is an alternative to access psychological factors.

This work presented a powerful application of the CNN technique for EEG signal classification. The model used in this study was able to understand most features that distinguish EEG signal acquired during consumption of beverage sweetened with caloric and non-caloric sweeteners. When we compare general performance with reference performance, mainly F_1_ score that consider recall and precision measures simultaneously we can deduce that misclassifications in sweeteners classes are greater than that in reference class.

Using a CNN for feature extraction has the advantage of not needing a prior filter model or featured engineered treatment as the kernel’s weights are obtained during training and time dependent features will be extracted by the internal structure of convolutional layer (42). However, DL demands an extensively large amount of data to achieve a well-behaved performance model, i.e., as the data increases, an extra well-behaved performance model can be achieved (43). This is a preliminary study, so we can improve the results of this study by increasing the number of samples tested per individual and also the number of individuals. This would allow us to train the network for each participant, for instance. However, this approach would involve the use of a taste delivery system to facilitate the increase in the number of samples.

This fact does not invalidate the main contribution of this study, which corroborates recent studies (5, 7, 14) stating that similar stimuli, despite being consciously indistinguishable, may result in different cortical responses.

We have compared brain signals acquired in response to the consumption of passion fruit juice sweetened with sucrose (caloric sweetener), sucralose, or aspartame (low-calorie sweeteners). We used the artifact-free data to feed a CNN. The results indicated that CNN received raw signal as input for multiclass classification and with supervised training was able to extract underling features and patterns from the signal with 0.823 accuracy. The main result presented here was precisely the CNN’s ability to analyse and classify brain signals obtained during tasting a sweetened beverage.

## Data availability statement

The datasets presented in this study can be found in online repositories. The names of the repository/repositories and accession number(s) can be found below: the EEG dataset that supports the findings of this study is available on GitHub at https://github.com/Atzingen/EEG_Sweetners_AI.

## Ethics statement

The studies involving human participants were reviewed and approved by the Ethics Committee of the Animal Science and Food Engineering College (FZEA) of the University of São Paulo (USP) (CAAE 59017516.6.0000.5422). The patients/participants provided their written informed consent to participate in this study.

## Author contributions

ACS, EC, and HA: conceptualization. NO and HA: data acquisition. ARS: data analysis. ACS, EC, and GA: design of methodology. GA: software development. ACS: writing – original draft, funding acquisition, and project administration. ACS, EC, GA, and HA: writing – review and editing. All authors contributed to the article and approved the submitted version.

## Conflict of interest

The authors declare that the research was conducted in the absence of any commercial or financial relationships that could be construed as a potential conflict of interest.

## Publisher’s note

All claims expressed in this article are solely those of the authors and do not necessarily represent those of their affiliated organizations, or those of the publisher, the editors and the reviewers. Any product that may be evaluated in this article, or claim that may be made by its manufacturer, is not guaranteed or endorsed by the publisher.

## Footnotes

1
https://github.com/Atzingen/EEG_Sweetners_AI
